# Comparison of point‐of‐care and central laboratory analyzers for blood gas and lactate measurements

**DOI:** 10.1002/jcla.22885

**Published:** 2019-03-29

**Authors:** Nuri Dyah Indrasari, Jessica Purwanti Wonohutomo, Ninik Sukartini

**Affiliations:** ^1^ Clinical Pathology Department, Faculty of Medicine, Cipto Mangunkusumo National Referral Hospital University of Indonesia Jakarta Indonesia

**Keywords:** blood gas, inter‐device variability, i‐STAT, lactate, Nova pHOx plus L

## Abstract

**Background:**

Blood gas analysis and blood lactate measurement have important roles in patient management. Point‐of‐care (POC) testing simplifies and provides rapid blood gas and lactate measurements. This study aimed to compare pH, pCO_2_, pO_2_, and lactate measurements between a POC device and a benchtop blood gas analyzer typically used in a hospital central laboratory, and to evaluate the inter‐device variability of the POC device.

**Methods:**

A cross‐sectional study was conducted with a sample size of 100. Each sample was measured for pH, pCO_2_, pO_2_, and lactate using a Nova pHOx plus L^®^ benchtop blood gas analyzer in the central laboratory and an i‐STAT^®^ handheld POC device. The results of both devices were compared using Pearson or Spearman correlation coefficients and Bland‐Altman tests. Testing of the inter‐device variability was done by using three different i‐STAT^®^ devices, and the results were compared statistically.

**Results:**

Strong correlations were observed for all test results. In Bland‐Altman analysis, ≥95% of the results were within the limits of agreement, with the exception of lactate, which had only 93%. The results that were beyond the limits were primarily lactate levels >8 mmol/L. Biases between the benchtop analyzer and the i‐STAT^®^ were not clinically significant, except pH. No significant inter‐device variability was observed between the i‐STAT^®^ analyzers.

**Conclusion:**

This comparison study of pH, pCO_2_, pO_2_, and lactate measurements between Nova pHOx plus L^®^ and i‐STAT^®^ analyzers showed good agreement. However, lactate measurement results >8 mmol/L on the i‐STAT^®^ analyzer should be interpreted with caution.

## 1 INTRODUCTION

Levels of blood gases and lactic acid are important for evaluating the equilibrium between tissue oxygen delivery and demand, as well as the acid‐base balance. Determining these levels is particularly essential in intensive care patient management, as blood gas and lactate levels are utilized as the basis for determining disease severity, evaluating treatments given, and estimating prognosis.^1^ According to the guidelines of the American Association for Respiratory Care (AARC; 2013), blood gas and lactate are measured to evaluate the acid‐base and oxygenation status of patients, the effectiveness of supplemental oxygen given, early goal‐directed therapy in sepsis, patient progress and patient circulation status.^1^ Thus, laboratory analyzers that provide rapid and accurate results are critically necessary.

Point‐of‐care (POC) blood gas and lactate analysis is an alternative choice that can reduce turnaround time, provide test results faster, and minimize pre‐analytical errors, which may interfere with the test. Currently, benchtop blood gas and lactate analyzers are generally available in hospital units, such as intensive care units (ICUs) and perinatology units, and are considered the gold standard for blood gas measurements.^2^ However, benchtop analyzers are not as portable are thus impractical for transport to another unit. Laboratory tests using handheld POC devices can be carried out near the patient in any location. Therefore, the availability of blood gas and lactate handheld POC devices should shorten examination time, so that the management of emergency and critical patients can proceed quickly. Nevertheless, it is crucial to compare handheld POC devices with benchtop analyzers to evaluate whether their respective measurements are indeed comparable.

The i‐STAT^®^ (Abbott point‐of‐care, Texas, USA) analyzer is a handheld blood gas and lactate POC analyzer that can deliver results quickly and be carried easily into any patient location in the hospital.^3^, ^4^ The aims of this study were to study the agreement of blood gas and lactate test results between an i‐STAT^®^ handheld POC device and a benchtop blood gas and lactate analyzer typically used in the central laboratory, and to determine whether there were any statistically or clinically significant biases between them. In addition, inter‐device variability of the i‐STAT^®^ handheld POC analyzer was also evaluated to determine whether different i‐STAT^®^ handheld devices would give significantly different test results.

## 2 MATERIALS AND METHODS

This study was performed in the central laboratory of Ciptomangunkusumo National Referral Hospital (Jakarta, Indonesia) between April and May 2017. The agreement of blood gas and lactate test results between the i‐STAT handheld device and the central laboratory benchtop blood gas analyzer (Nova pHOx plus L^®^, Nova Biomedical, Massachusetts, USA) was studied using 100 arterial blood samples sent for blood gas and lactate measurement to the central laboratory. The Clinical Laboratory Standards Institute (CLSI) guidelines recommend a minimum of 40 observations in method comparison studies, as stated in the CLSI Guidelines for Method Comparison and Bias Estimation Using Patient Samples (NCCLS document EP9‐A2).^5^ Analytical performances of the i‐STAT^®^ and Nova pHOx plus L^®^ were also evaluated for precision. Inter‐device variability of the i‐STAT^®^ analyzers was also assessed by measuring three levels of control solution on three different i‐STAT^®^ devices.

Arterial blood samples were collected from patients in the inpatient wards and ICUs in 3 cc blood gas syringes and were analyzed first on the central laboratory blood gas analyzer, the Nova pHOx plus L^®^, and immediately thereafter on the i‐STAT^®^ analyzer. Exclusion criteria included specimen volumes <500 µL. Blood gas and lactate measurements required 125 μL specimen for the Nova pHOx plus L^®^ and 95 μL for the i‐STAT^®^ analyzer.^3^, ^4^ Specimens were analyzed on the i‐STAT^®^ analyzer using CG4+ disposable cartridges. The analytes being studied for blood gas were pH, pCO_2_, and pO_2_ because other blood gas parameters (eg, total CO_2_, HCO_3_, Base Excess, and sO_2_) are calculated based on the three aforementioned parameters on the i‐STAT^®^ analyzer.^3^

Within‐run and between‐day precision tests for i‐STAT^®^ and Nova pHOx plus L^®^ analyzers were measured using three levels of control solutions. Within‐run tests were performed 10 times for each level of control solution, while between‐day tests were performed for 10 consecutive days. The results were then compared with precision data stated by the manufacturer. The inter‐device variability of the i‐STAT^®^ was evaluated with ANOVA or Kruskal‐Wallis tests.

The correlation between the results from the i‐STAT^®^ POC device and the Nova pHOx plus L^®^ analyzer was determined using Pearson and Spearman correlation coefficients. In addition, Bland‐Altman analysis was used to measure the level of agreement between the i‐STAT^®^ and the Nova pHOx plus L^®^ based on the values of bias and limits of agreement. Bland‐Altman plots were made by plotting the differences between analyzers against the calculated means. The Passing‐Bablok regression test was performed to obtain the pH, pCO_2_, pO_2_, and lactate prediction equations to see whether there were constant or proportional differences in pH, pCO_2_, pO_2_, and lactate test results between the handheld and benchtop analyzers.^6^

To assess whether the analytical differences between the i‐STAT^®^ and Nova pHOx plus L^®^ may lead to clinically significant discrepancies, the values of bias between the analyzers were compared to acceptable analytical performance based on the 1992 Clinical Laboratory Improvement Amendments (CLIA) proficiency testing criteria target value for pH ± 0.04 pH units, for pCO_2_ ± 5 mm Hg, and for pO_2_ ± 3 SD.^7^ An acceptable analytical performance for lactate was based on the 2014 Royal College of Pathologists of Australasia (RCPA) Allowable Limits of Performance, with a target value of ±1.0 mmol/L for lactate ≤10.0 mmol/L.^8^ Discrepancies between the values obtained by the i‐STAT^®^ and the Nova pHOx plus L^®^ were not considered clinically significant if they were less than the target value criteria of acceptable analytical performance.

## 3 RESULTS

Results of the within‐run and between‐day imprecision study of the i‐STAT^®^ and Nova pHOx plus L^®^ analyzers are summarized in Table 1. The precision profile of each analyzer for pH, pCO_2_, pO_2_, and lactate derived from this study showed comparable results to the claims by each manufacturer. In general, the precision profile of the i‐STAT^®^ and the Nova pHOx plus L^®^ was considered equivalent.

**Table 1:** Imprecision study results

Analyzer	i‐STAT^b^			NOVA pHOx plus L^c^		
Control solution level	1	2	3	1	2	3
Within‐run CV (%)^a^						
pH	0.05 (0.07)	0.05 (N/A)	0.04 (0.04)	0.13 (N/A)	0.12 (N/A)	0.11 (N/A)
pCO_2_	1.26 (2.50)	1.53 (N/A)	1.86 (2.00)	1.78 (3.00)	1.49 (3.00)	2.22 (3.00)
pO_2_	4.60 (4.80)	4.23 (N/A)	2.19 (4.10)	0.46 (3.00)	0.28 (3.00)	0.65 (3.00)
Lactate	0.62 (1.30)	0.81 (N/A)	1.20 (3.70)	0.83 (3.00)	1.52 (3.00)	4.58 (3.00)
Between‐day CV (%)^a^						
pH	0.07 (0.07)	0.04 (N/A)	0.04 (0.04)	0.15 (N/A)	0.11 (N/A)	0.10 (N/A)
pCO_2_	2.09 (2.50)	1.16 (N/A)	1.37 (2.00)	2.85 (5.00)	3.10 (5.00)	2.19 (5.00)
pO_2_	4.98 (4.80)	2.52 (N/A)	1.91 (4.10)	2.25 (5.00)	0.91 (5.00)	2.91 (5.00)
Lactate	0.79 (1.30)	0.56 (N/A)	1.99 (3.70)	3.26 (6.00)	3.06 (6.00)	4.98 (6.00)

N/A, Not available.

a Values in bracket refer to CV claimed by the manufacturer.

b The manufacturer did not differentiate between CV of within‐run and CV of between‐day.

c The manufacturer did not differentiate between CV of each level.

Inter‐device variability tests demonstrated no significant variability between the three i‐STAT^®^ analyzers used in this study. There was no *P*‐value below 0.05 in the inter‐device variability tests, meaning no significant differences between the test results of the three analyzers were detected. The *P*‐value of the inter‐device variability tests is shown in Table 2.

**Table 2:** Inter‐device variability test results between three i‐STAT analyzers

Level	pH	pCO_2_	pO_2_	Lactate
1	*P* = 0.497	*P* = 0.903	*P* = 0.773	*P* = 0.650
2	*P* = 0.577	*P* = 0.204	*P* = 0.604	*P* = 0.365
3	*P* = 0.368	*P* = 0.096	*P* = 0.652	*P* = 0.405

Strong correlation coefficients (r) between the test results of the i‐STAT^®^ and the Nova pHOx plus L^®^ were observed in the comparison study—pH (*r* = 0.893), pCO_2_ (*r* = 0.843), pO_2_ (*r* = 0.983), and lactate (*r* = 0.986); *P* < 0.001. The results of the Passing‐Bablok regression test performed between the i‐STAT^®^ and the Nova pHOx plus L^®^ are shown in Table 3. There was no constant nor proportional bias observed between the analyzers for pH, while there was a constant bias for pCO_2_, a proportional bias for lactate, and both for pO_2_.

**Table 3:** Passing‐Bablok regression analysis

Parameter	Passing‐Bablok regression^a^	Intercept (95% CI)	Slope (95% CI)	Cusum test for linearity
pH	*y* = −0.399 + 1.048 *x*	−1.230 to 0.388	0.942‐1.161	*P* = 0.85
pCO_2_	*y* = 4.325 + 0.912 *x*	0.470‐8.157	0.819‐1.010	*P* = 0.96
pO_2_	*y* = −3.037 + 1.056 *x*	−4.628 to (−1.239)	1.015‐1.100	*P* = 0.53
Lactate	*y* = −0.035 + 0.871 *x*	−0.193 to 0.122	0.825‐0.929	*P* = 0.70

a *y* = i‐STAT analyzer, *x* = Nova pHOx plus L analyzer.

**Figure 1:**
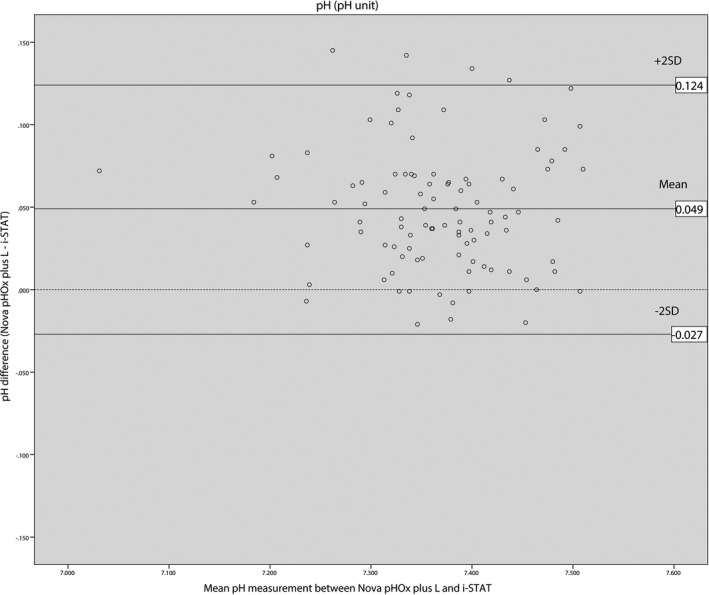
Bland‐Altman plot for pH

**Figure 2:**
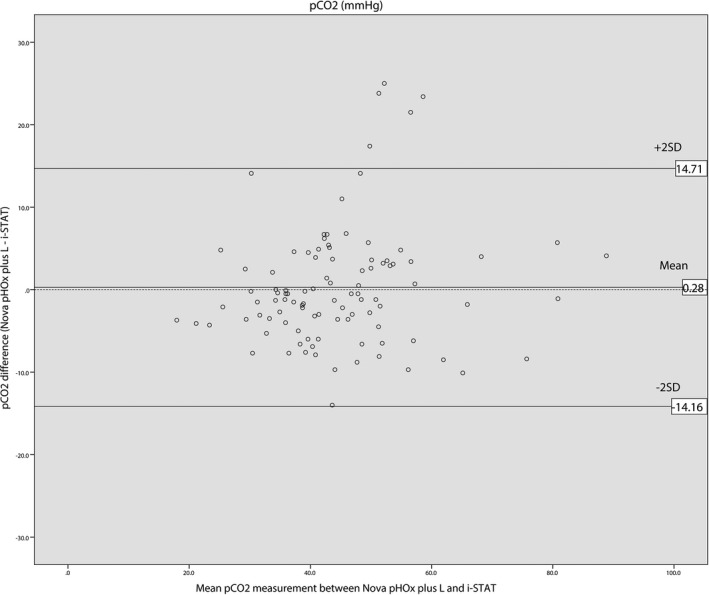
Bland‐Altman plot for pCO_2_

**Figure 3:**
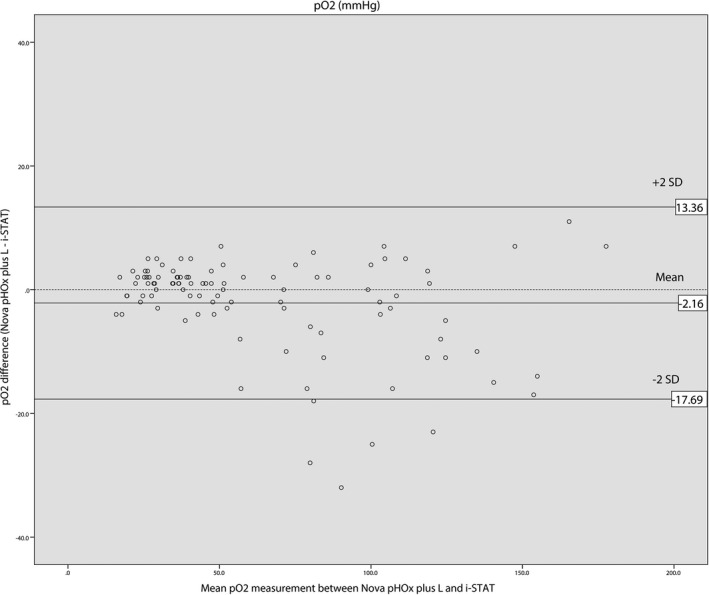
Bland‐Altman plot for pO_2_

**Figure 4:**
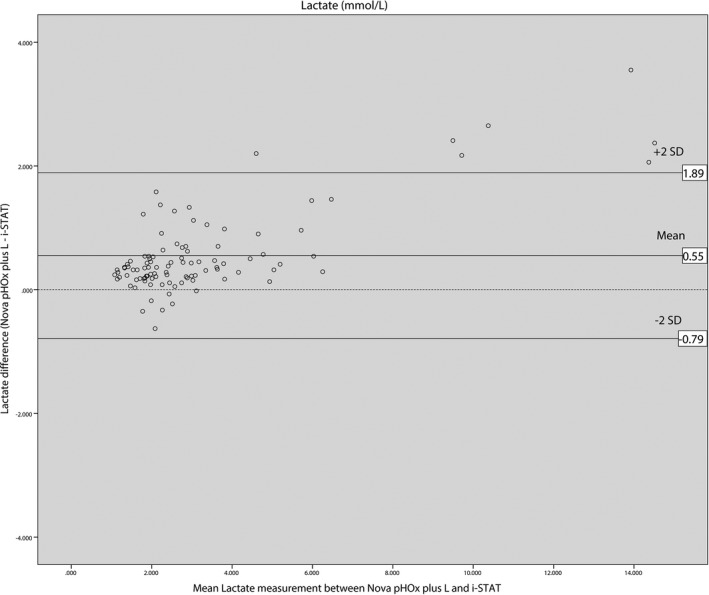
Bland‐Altman plot for lactate

Bland‐Altman analysis of pH results displayed the average bias for the i‐STAT^®^ analyzer of 0.049 pH units lower than the Nova pHOx plus L^®^ with the 95% limits of agreement ranging from −0.027 to 0.124. The average bias of pCO_2_ for the i‐STAT^®^ was 0.28 mm Hg lower than the Nova pHOx plus L^®^ with 95% limits of agreement ranging from −14.16 to 14.71. The average bias of pO_2_ was 2.16 mm Hg higher on the i‐STAT^®^ compared with the Nova pHOx plus L^®^ with 95% limits of agreement ranging from −17.69 to 13.36. The average bias of lactate for the i‐STAT^®^ was 0.55 mmol/L lower than the Nova pHOx plus L^®^ with 95% limits of agreement ranging from −0.79 to 1.89. Bland‐Altman plots showed that 96% of the pH differences were within the limits of agreement, with 95% for both pCO_2_ and pO_2_ differences, but only 93% for lactate differences. Seven out of eight lactate differences outside the limits of agreement were higher than 8 mmol/L. Bland‐Altman plots obtained in this study are shown in Figures 1, 2, 3, 4.

The mean biases of pCO_2_, pO_2_, and lactate measurements between the i‐STAT^®^ and the Nova pHOx plus L^®^ analyzers were not considered clinically important according to acceptable analytical performance based on the 1992 CLIA Proficiency Testing criteria and the 2014 RCPA Allowable Limits of Performance target value.^7^, ^8^ The mean biases obtained in this study were 0.28 mm Hg for pCO_2_ (target value ± 5 mm Hg), −2.16 mm Hg for pO_2_ (target value ± 3 SD, SD: 7.76 mm Hg), and 0.55 mmol/L for lactate pO_2_ (target value ± 3 SD), and 0.55 mmol/L (target value was ± 1.0 mmol/L). The bias of pH measurement was 0.049, which was slightly higher than the target value of 0.04.

## 4 DISCUSSION

Blood gas and lactate tests are frequently requested laboratory tests for ICU and emergency patients to evaluate acid‐base status, ventilation, and arterial oxygenation sufficiency. Sudden changes in those parameters may indicate patient deterioration and life‐threatening situations. Hence, rapid results are frequently required for effective patient management. Arterial blood gas analysis consists of three separate measurements—pH, pCO_2_, and pO_2_; however, depending on the analyzer this may also include other calculated parameters, such as bicarbonate, base excess, total carbon dioxide, and blood oxygen saturation.^2^ The gold standard method for blood gas measurement according to the International Federation of Clinical Chemistry (IFCC) guideline is direct ion selective electrode (ISE).^9^ Currently, most blood gas analyzers use the ISE method with a triple electrode system to measure pH, pCO_2_, and pO_2_, including the i‐STAT^®^ and Nova pHOx plus L^®^ analyzers.

POC testing is laboratory testing performed at or near the site of patient care without the need for sophisticated laboratory equipment.^10^ There are two types of blood gas and lactate POC devices: small bench top analyzers and handheld instruments. Handheld blood gas and lactate analyzers, the use of which is rapidly increasing, are more compact and portable compared with the gold standard benchtop analyzer that is commonly available in hospital units. However, the availability of the different types of blood gas and lactate analyzers within a hospital may raise clinician concerns about the interchangeability of the measurement results and the performance of the handheld POC analyzer.

The within‐run tests on the i‐STAT^®^ analyzer demonstrated a coefficient of variation (CV) <5% for blood gas and lactate when control materials were run 10 times covering three level of concentrations. Between‐day precision tests of the i‐STAT^®^ analyzer in this study demonstrated similar results. The precision test CVs acquired in this study were higher than in the study by Leino et al (2011); the CV of which was <3% for blood gas and lactate.^11^ Karon et al (2007) reported that the CV of lactate precision tests using the i‐STAT^®^ analyzer was 3%.^12^ However, the CV of the i‐STAT^®^ analyzer obtained in this study was less than the manufacturer CV of blood gases and lactate, except for level 1 pO_2_ between‐day test.^3^ Precision test CVs of the Nova pHOx plus L^®^ in this study displayed similar results, with CV < 5% for blood gases and lactate.

Inter‐device variability test of the i‐STAT^®^ analyzer displayed no significant variability between the three i‐STAT^®^ devices used in the study. This result confirms that the usage of multiple i‐STAT^®^ analyzers in the hospital would not give significantly different blood gas and lactate measurement results. To the best of our knowledge, there has been no previous study investigating the inter‐device variability of i‐STAT^®^ handheld analyzers.

Blood gas and lactate measurement results in this study displayed strong correlation between the i‐STAT^®^ device and the Nova pHOx plus L^®^ benchtop analyzer. Correlation coefficients are the statistical comparison of the degree of linear relationship between two variables, in this case blood gas analysis and lactate results between the two analyzers.^13^ Previous studies by Leino et al (2011) and Karon et al (2007) compared the i‐STAT^®^ POC handheld device to central laboratory analyzers, which also displayed strong correlation between the analyzers.^11^, ^12^ Passing‐Bablok regression test revealed that there was neither proportional nor constant difference for pH results between the two analyzers since the value zero was included in the 95% CI for intercept and value one was included in the 95% CI for slope, while other parameters displayed variable results.^6^ Partial O_2_ pressure measurement results revealed that there were both proportional and constant differences between the analyzers, while partial CO_2_ pressure and lactate measurement results displayed proportional and constant differences between the two analyzers, respectively. The equations derived from the Passing‐Bablok regression test may be useful for predicting measurement differences between the i‐STAT^®^ and Nova pHOx plus L^®^, in view of the fact that blood gas and lactate values derived from the i‐STAT^®^ handheld analyzer and benchtop laboratory analyzer are frequently used interchangeably by clinicians.

Bland‐Altman plots showed that 95% of the differences were between the range of the limits of agreement for pH, pCO_2_, and pO_2_, while for lactate it was only 93% of the differences were between limits of agreement. Discrepancies between the i‐STAT^®^ and the Nova pHOx plus L^®^ were more significant for lactate values of more than 8 mmol/L (Figure 4). Lactate values measured on the i‐STAT^®^ analyzer were lower than on the Nova pHOx plus L^®^. Karon et al (2007) had previously compared lactate measurements between the i‐STAT^®^ and core laboratory analyzer (Vitros 250^®^).^12^ In that study, discrepancies were more significant at values >6 mmol/L, with i‐STAT^®^ results being lower than those of the Vitros 250^®^.^12^ Leino et al (2011) reported that i‐STAT^®^ analyzers gave lower lactate values compared with core laboratory plasma analyzers, especially with high lactate concentrations.^11^ The same tendency was observed by Ismail et al (2015) in their study comparing the i‐STAT^®^, GEM Premier 4000^®^, and OMNI S^®^ analyzers.^14^ Therefore, clinicians should be informed that caution must be used when comparing high lactate values between the i‐STAT^®^ device and central laboratory analyzers.

Clinically significant bias was observed for pH values measured with the i‐STAT^®^ analyzer since they were slightly higher than the target value of acceptable analytical performance. In spite of this, the majority of the results were within acceptable limits. However, any pH value that was near the upper or lower reference limit should be interpreted with caution.^7^ Information regarding patient clinical condition, medical history, and treatments should be determined whenever possible.

In conclusion, the differences in blood gas (pH, pCO_2_, pO_2_) and lactate values among the i‐STAT^®^ POC analyzer and benchtop blood gas laboratory analyzer are negligible. However, medical facilities that use both the i‐STAT^®^ analyzer and central laboratory benchtop blood gas analyzers should inform clinicians that caution must be used when comparing high lactate values between point‐of‐care and benchtop blood gas laboratory measurement results. There was no inter‐device variability observed between different i‐STAT^®^ devices, thus measurement results from multiple i‐STAT^®^ analyzers can be employed interchangeably in daily practice.
